# Effect of Functional Inhibition of BACE1 on Sensitization to γ-Irradiation in Cancer Cells

**DOI:** 10.3390/cimb46010028

**Published:** 2024-01-02

**Authors:** Keitaro Nakamoto, Sota Kikuhara, Hiroaki Fujimori, Barkha Saraswat, Zhongming Gao, Ankitha Vadi Velu, Zongxiang Zhang, Ying Tong, Shoji Imamichi, Tadashige Nozaki, Yasufumi Murakami, Mitsuko Masutani

**Affiliations:** 1 Department of Molecular and Genomic Biomedicine, Center for Bioinformatics and Molecular Medicine, Nagasaki University Graduate School of Biomedical Sciences, 1-12-4, Sakamoto, Nagasaki 852-8523, Japan; bb55317803@ms.nagasaki-u.ac.jp (K.N.); bb55322032@ms.nagasaki-u.ac.jp (B.S.); bb55323014@ms.nagasaki-u.ac.jp (Z.G.); bb55322801@ms.nagasaki-u.ac.jp (A.V.V.); bb55323025@ms.nagasaki-u.ac.jp (Z.Z.); y-tong@nagasaki-u.ac.jp (Y.T.); nozaki@cc.osaka-dent.ac.jp (T.N.); 2 Division of Chemotherapy and Clinical Cancer Research, National Cancer Center Research Institute, 5-1-1 Tsukiji, Chuo-ku, Tokyo 104-0045, Japan; 3 Department of Biological Science and Technology, Faculty of Industrial Science and Technology, Tokyo Univsersity of Science, 2641 Yamazaki, Noda 278-8510, Chiba, Japan; yasufumi@rs.noda.tus.ac.jp; 4 Department of Pharmacology, Faculty of Dentistry, Osaka Dental University, Osaka 573-1121, Japan

**Keywords:** BACE1, γ-H2AX, γ-irradiation, radiosensitization, cancer cell

## Abstract

Developing strategies for the radiosensitization of cancer cells by the inhibition of genes, which harbor low toxicity to normal cells, will be useful for improving cancer radiotherapy. Here, we focused on a β-site of amyloid precursor protein (APP)-cleaving enzyme 1 (BACE1; β-secretase, memapsin-2). By functional inhibition of this peptidase by siRNA, it has also recently been shown that the DNA strand break marker, γH2AX foci, increased, suggesting its involvement in DNA damage response. To investigate this possibility, we knocked down *BACE1* with siRNA in cancer cell lines, and sensitization to γ-irradiation was examined by a colony formation assay, γH2AX foci and level analysis, and flow cytometry. *BACE1* knockdown resulted in the sensitization of HeLa, MDA-MB-231, U2OS, and SAOS cells to γ-irradiation in a diverse range. *BACE1* knockdown showed a weak radiosensitization effect in osteosarcoma U2OS cells, which has a normal p53 function. HeLa and SAOS cells, which harbor p53 dysfunction, exhibited a greater level of radiosensitization. These results suggest that BACE1 may be a potential target for the radiosensitization in particular cancer cells.

## 1. Introduction

Establishing the strategies for biological radiosensitization is expected for cancer radiotherapy. Radiosensitizing effects of inhibitors for DNA repair responses, including Chk1 [1], poly(ADP-ribose) polymerase [2], poly(ADP-ribose) glycohydrolase [3,4], and heat-shock protein 90 [5], have been reported, and some of these inhibitors are being evaluated in clinical trials. Because cancer cells harbor various types of mutations or aberrations in cellular pathways, diverse strategies for radiosensitization should be necessary. When we screened radiosensitization targets using shRNA library screening, we found that the β-site of amyloid precursor protein (APP)-cleaving enzyme 1 (BACE1; β-secretase, memapsin-2) was picked up as a unique candidate of radiosensitization target groups [6].

It is known that sialyltransferases have intrinsic roles in cancer cell growth and resistance to cancer therapies. Some sialyltransferases are overexpressed in cancers, including ovarian and colon cancers [7]. However, the radiosensitization effects of sialyltransferase inhibitors have not been well studied. BACE1 is one of two secretases that cleave APP to produce an extracellular isoform (sAPPβ) [8]. With another secretase, PS/γ-secretase, BACE1 has been studied as a target for the treatment of Alzheimer’s disease [9]. BACE1 was also demonstrated to regulate the Na_v_ channel and metabolism in neuronal cells in the brain and K_v_ channel dynamics in neuronal and non-neuronal cells, including in the heart and brain [10,11]. On the other hand, BACE1 belongs to the peptidase A1 protein family and has a function as a processing enzyme for sialyltransferase in the Golgi apparatus [12]. *BACE1* knockout mice have been produced but showed no apparent phenotypes, suggesting that BACE1 inhibition will not cause severe side effects [13,14,15]. Specific BACE1 inhibitors have also been developed, and some of the inhibitors have been evaluated in clinical trials of Alzheimer’s disease [16,17,18,19,20].

Meanwhile, it has been also reported that BACE1 inhibition potentiated γH2AX as a DNA double-strand break marker, suggesting its direct or indirect involvement in DNA repair response [21]. Here, in this study, we investigated the radiosensitization effect of BACE1 functional inhibition by gene knockdown in human cancer cells. Our results suggest that BACE1 could be a candidate for radiosensitization in certain cancer cells.

## 2. Materials and Methods

### 2.1. Cell Culture and Materials

T-Rex HeLa cells (Gibco Life Technologies Corp., Carlsbad, CA, USA) and breast cancer MDA-MB-231 (ATCC, Manassas, VA, USA) were cultured as described by the manufacturers. Briefly, the cells were cultured with an MEM medium (Gibco Life Technologies Corp.) containing 10% fetal bovine serum (HyClone, Cytiva, Tokyo, Japan) and penicillin-streptomycin (Gibco Life Technologies Corp.). Osteosarcoma U2OS (ATCC) and SAOS (ATCC) were cultured with McCoy’s 5A medium containing 10% fetal bovine serum (HyClone) and penicillin-streptomycin (Gibco Life Technologies Corp.). WI-38 cells (Meiji-Seika Pharma Co., Tokyo, Japan) were cultured with an MEM medium (Gibco Life Technologies Corp.) containing 10% fetal bovine serum (HyClone) and penicillin-streptomycin (Life Technologies). The BACE1 inhibitor KMI-1303 was obtained from Fuji WAKO Chemicals (Osaka, Japan).

### 2.2. RNAi Experiments

Silencer Select Validated siRNA targeting *BACE1* (s24218, Integrated DNA Technologies, Coralville, IA, USA) was transfected by Hiperfect (Qiagen, Hilden, Germany) or Lipofectamine RNAiMAX (Thermo Fisher Scientific, Waltham, MA, USA) based on the manufacturer’s protocol. Briefly, 3 × 10^5^ cells were seeded to a well of 6-well culture plates (Thermo Fisher Scientific) for 24 h before transfection. Then, the cells were treated with the transfection reagent with the All Stars Negative Control siRNA (Qiagen) as a non-silencing control siRNA.

### 2.3. Gene Expression Analysis

Forty-eight hours after the transfection of *BACE1* siRNA, RNA was harvested by a High Pure RNA Isolation Kit (Roche, Basel, Switzerland), and 2 μg of purified total RNA was reverse-transcribed by a High Capacity RNA-to-cDNA Kit (Applied Biosystems, Waltham, MA, USA), as described by the manufacturers. Real-time RT-PCR was performed as previously described [22]. The forward primer sequence for *BACE1* was TGCCCCGGGAGACCGACGAAG and the reverse was GGGGGCAGCACCCACTGCAAAG.

### 2.4. γ-Irradiation

Exponentially growing cells were irradiated by an ^137^Cs γ-irradiator (Gammacell 220, Nordion, Ottawa, ON, Canada) at a dose rate of 1.0 Gy/min at the National Cancer Center Research Institute and Nagasaki University (PS-3100SE, Pony Industry, Osaka, Japan).

### 2.5. Cell Proliferation Assay

Four days after γ-irradiation, cultured cells were detached and scattered by accutase treatment. Then, the live cell number was counted by a TC-10 automated cell counter (Bio-rad) and a modified MTT (methyl thiasolyl tetrazolium) assay (CCK Assay, Dojindo, Kumamoto, Japan) with biological replicates of *n* = 3.

### 2.6. Clonogenic Survival Assay

A clonogenic survival assay was performed as previously described [2] with some modifications. Cells were seeded in 6-well culture dishes 12 h before irradiation with biological replicates of *n* = 3. Nine days after seeding, colonies were fixed with 10% neutralized formalin (Fuji WAKO Chemicals) and stained with 0.1% crystal violet solution. Then, colonies composed of more than approximately 50 cells were counted.

### 2.7. Flow Cytometry

Cells were trypsinized and fixed with 70% ethanol. After rinsing with PBS, cells were treated with a staining solution (200 ng/mL RNase A and 20 μg/mL propidium iodide in PBS) for 1 h at room temperature and analyzed by fluorescence-activated cell sorting (FACS) using a FACS Calibur system (Beckton and Dickinson, Franklin Lakes, NJ, USA) with independent replicates of *n* = 3. Data acquisition was performed with CellQuest CellQuest Pro ver. 5.2.1 (Becton Dickinson) software.

### 2.8. γH2AX Foci Detection and Immunoblot Analysis

For γH2AX foci detection, cells were fixed with 4% paraformaldehyde and subsequently with methanol for 10 min at −20 °C. After permeabilization and blocking, the slides were incubated with anti-γ-H2AX (Cell Signalling Technology Danvers, MA, USA) and Alexa594-conjugated secondary antibody with 4′,6-diamidino-2-phenylindole (DAPI) staining. For immunoblot analysis, cells were lysed with Laemmli’s buffer and then sonicated, and protein concentration was measured by a Protein Assay (Bio-Rad, Hercules, CA, USA). Proteins were then subjected to electrophoresis on an SDS-polyacrylamide gel followed by transfer to a Sequi-Blot^TM^ PVDF membrane (Bio-Rad) as described elsewhere. Immunoblot analysis was carried out using the following primary antibodies: anti-γ-H2AX (1:1000; Millipore, Burlington, MA, USA), anti-PARP-1 (1:1000; Cell Signalling Technology), and anti-β-actin (1:50,000, Sigma-Aldrich, St. Louis, MI, USA). The secondary antibodies were horseradish peroxidase-linked immunoglobulin, and immune complexes were detected using an enhanced chemiluminescence reaction kit (Millipore).

### 2.9. Statistical Analysis

Statistical analysis was carried out by Tukey’s test using JMP Pro 17 (SAS Institute Inc., Cary, NC, USA).

## 3. Results

### 3.1. Radiosensitization Effect of BACE1 Knockdown and Its Expression Level in Cancer Cells

**Figure 1:**
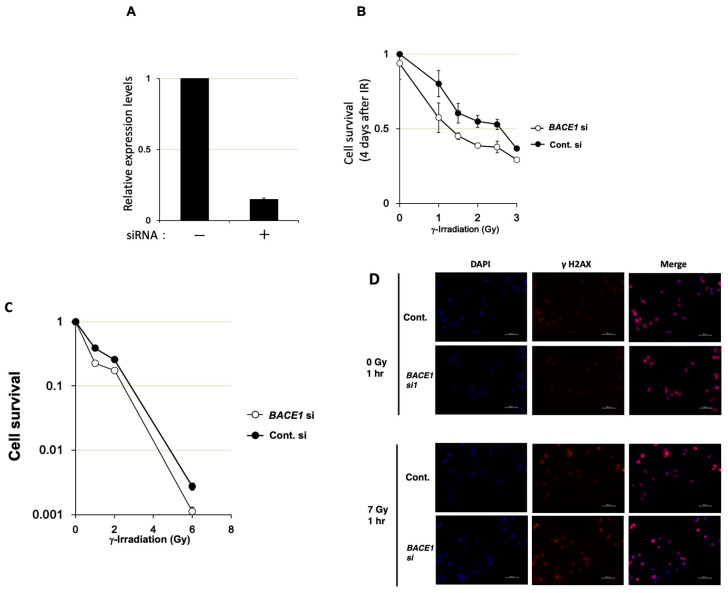
Effect of *BACE1* knockdown on T-Rex HeLa cell growth after γ-irradiation. (**A**) Real-time PCR analysis of *BACE1* mRNA expression 2 days after the transfection of siRNA for *BACE1* and control for siRNA. The relative expression level was calculated by the ratio of the expression levels of *BACE1* and *GUSB* genes. Bars, mean ± SE. (**B**) The results of the modified MTT assay 4 days after γ-irradiation with biological replicates of *n* = 3. Two days after the transfection of siRNAs, cells were γ-irradiated. Mean ± SE was plotted. (**C**) The effect of *BACE1* knockdown on the clonogenic survival in T-Rex HeLa cells after γ-irradiation. (**D**) γ-H2AX foci detection was carried out after *BACE1* siRNA transfection. One hour after γ-irradiation at 7 Gy or mock irradiation, the cells were fixed, and γ-H2AX foci (red) were observed with nuclear staining with DAPI (blue). Bars, 50 μm.

*BACE1* siRNA was transfected into T-Rex HeLa cells. The real-time PCR analysis showed that *BACE1* mRNA expression was reduced to nearly 15% of the control level two days after transfection (Figure 1A). The MTT assay revealed that *BACE1* knockdown did not show cytotoxicity. On the other hand, *BACE1* knockdown caused the suppression of cell growth from a low dose, namely at the range of 1–3 Gy (Figure 1B). In the clonogenic survival assay, *BACE1* knockdown resulted in sensitization of T-Rex HeLa cells to γ-irradiation with an enhancement ratio of 1.2 at 10% survival (Figure 1C). We also analyzed γ-H2AX foci detection, as shown in Figure 1D, and observed a higher intensity 1 h after 7 Gy irradiation in the *BACE1* knockdown compared to the contol (53% vs. 32%, respectively, whereas the values were less than 12% in unirradiated conditions of both the knockdown and control).

**Figure 2:**
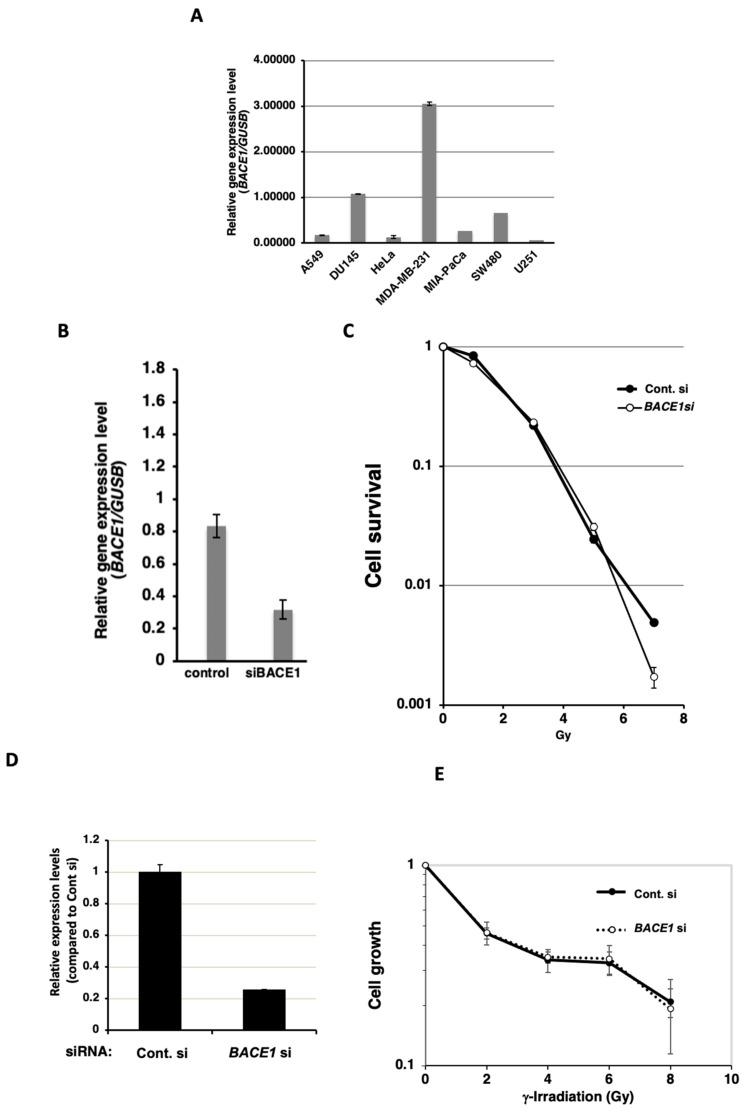
Expression levels of *BACE1* in cancer cell lines and the effect of *BACE1* knockdown in WI-38 and MDA-MB-231 cells after γ-irradiation. (**A**) Real-time PCR analysis of *BACE1* mRNA expression 2 days after the transfection of siRNA for *BACE1* and control for siRNA with biological replicates of *n* = 3. The relative expression level was calculated by the ratio of expression levels of *BACE1* and *GUSB* genes. Bars, mean ± SE. (**B**) The effect of *BACE1* knockdown in MDA-MB-231 cells after γ-irradiation. (**C**) The effect of *BACE1* knockdown on clonogenic survival in MDA-MB-231 cells after γ-irradiation with biological replicates of *n* = 3. (**D**) Real-time PCR analysis of *BACE1* mRNA expression in WI-38 cells 2 days after the transfection of siRNA for *BACE1* and control with biological replicates of *n* = 3. The relative expression level was calculated by the ratio of the expression levels of *BACE1* and *GUSB* genes. Bars, mean ± SE. (**E**) The results of the modified MTT assay after γ-irradiation in WI-38 cells with biological replicates of *n* = 3. Two days after the transfection of siRNAs, cells were γ-irradiated. Mean ± SE was plotted.

We further analyzed the expression level of *BACE1* in various cancer cell lines. As shown in Figure 2A, the cancer cell lines showed diverse levels of *BACE1* expression levels. Breast cancer MDA-MB-231 cells showed the highest level among the analyzed cell lines. Therefore, we analyzed the radiosensitization effect of *BACE1* knockdown in MDA-MB-231 cells by a colony formation assay. The *BACE1* knockdown exhibited a limited radiosensitization effect at a high dose of 7 Gy but not at the lower doses.

We then investigated whether *BACE1* knockdown radiosensitizes normal human cell lines. For this purpose, we used a normal human lung fibroblast cell line, WI-38, as a normal tissue surrogate. Because the colony formation ability of WI-38 was found to be low, we examined cell growth by an MTT assay. In WI-38, *BACE1* knockdown to approximately 25% of the control level was observed at the mRNA level (Figure 2D). The plating efficiency in *BACE1* knocked down cells was approximately 86%. As shown in Figure 2E, *BACE1* knockdown did not radiosensitize WI-38 cells.

### 3.2. Radiosensitization Effect of BACE1 Knockdown and BACE1 Inhibitor in U2OS Cells

**Figure 3:**
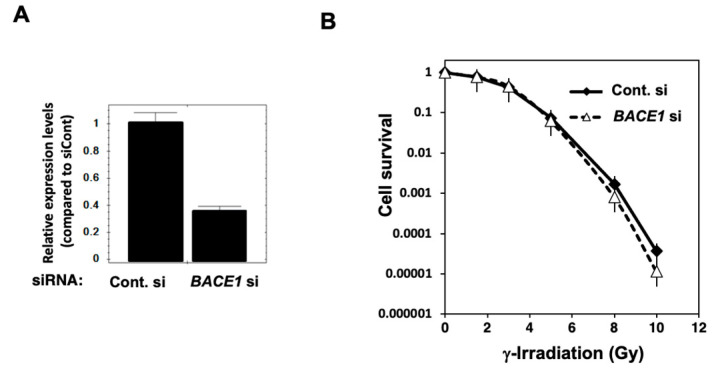

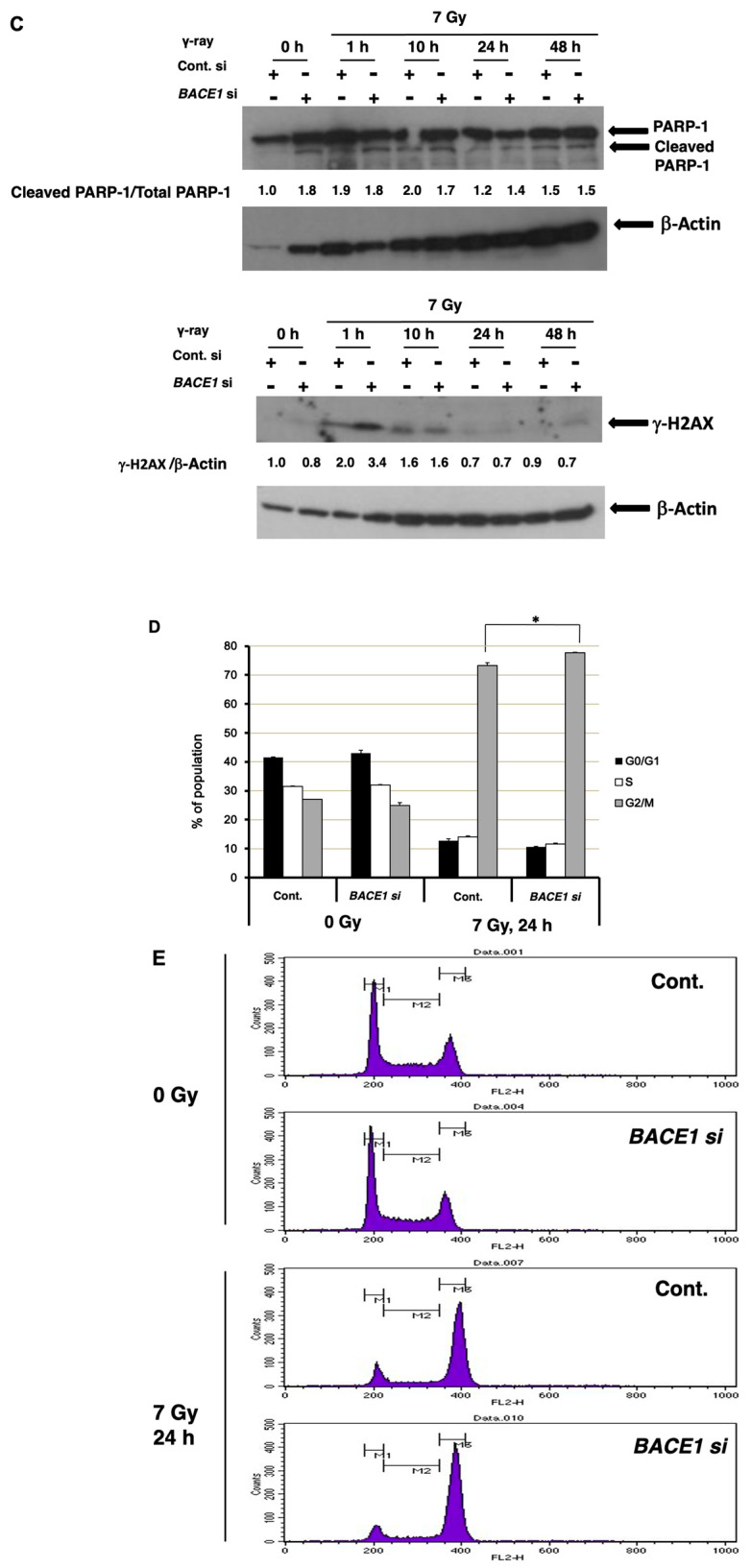
The effect of *BACE1* knockdown in U2OS cells after γ-irradiation. (**A**) The expression level of *BACE1* mRNA analyzed by real-time PCR analysis two days after the transfection of siRNAs. The relative expression level was calculated by the ratio of the expression levels of *BACE1* and *GUSB* genes with biological replicates of *n* = 3. Bars, mean ± SE. (**B**) A clonogenic survival assay after γ-irradiation was carried out, as described in the Materials and Methods, with biological replicates of *n* = 3. (**C**) Western blot analysis after γ-irradiation at 7 Gy for apoptotic marker PARP1 cleavage and γ-H2AX levels. Representative data were shown. (**D**) The effect of *BACE1* knockdown on cell cycle distribution in U2OS cells after γ-irradiation. *, *p* < 0.05. Percentages of cell cycle phase 24 h after 7 Gy γ-irradiation with independent replicates of *n* = 3. (**E**) Representative propidium iodide (PI) staining patterns without irradiation and 24 h after irradiation.

**Figure 4:**
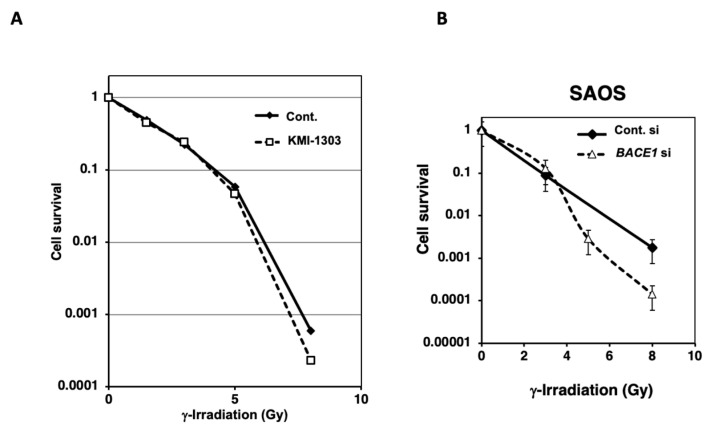
The effect of BACE1 inhibitors in U2OS cells and *BACE1* knockdown in SAOS cells after γ-irradiation. (**A**) U2OS cells were pretreated with BACE1 inhibitor KMI-1303 at 10 μM, and a clonogenic survival assay after γ-irradiation was carried out with biological replicates of *n* = 3. (**B**) A clonogenic survival assay in *BACE1* knocked down and control siRNA treated SAOS cells after γ-irradiation was carried out with biological replicates of *n* = 3.

The radiosensitization effect could be different depending on the genetic and epigenetic states of cancer cells. T-Rex HeLa cells show inactivation of tumor suppressor p53. Here, the *BACE1* knockdown effect has also been examined in osteosarcoma U2OS cells, which have normal p53 function. In U2OS cells, the mRNA expression of *BACE1* was reduced to nearly 40% of the control level (Figure 3A). A radiosensitization effect was not observed at 10% survival but was observed at a higher dose of 6–10 Gy by a clonogenic survival assay (Figure 3B). A weak cytotoxic effect was observed with U2OS cells by *BACE1* knocked down alone; the cleaved form of apoptosis-associated cleavage of PARP-1 was detected by Western blot analysis (Figure 3C, upper panel). As mentioned earlier, the increase in γ-H2AX foci, a DNA strand break marker, is reported in HeLa cells after *BACE1* knockdown [21]. In T-Rex HeLa cells, we observed that a higher level of γ-H2AX foci was present 1 h after 7 Gy irradiation. After γ-irradiation, in parallel to the phosphorylation of H2AX (detected as γ-H2AX), acetylation and stabilization can occur, and because BACE1 is known as a peptidase, we analyzed the dynamic state of γ-H2AX by Western blot analysis in U2OS cells. We observed an augmented transient level of γ-H2AX after γ-irradiation under *BACE1* knockdown (Figure 3C, lower panel). An apoptosis marker, PARP1 cleavage, was only slightly higher under the *BACE1* knocked-down condition. Flow cytometric analysis at 24 h after 7 Gy irradiation showed that *BACE1* knockdown slightly augmented G2/M arrest (*p* < 0.05) after γ-irradiation (Figure 3D,E). We also analyzed whether the BACE1 inhibitor KMI-1303 showed radiosensitization effects in U2OS cells. As shown in Figure 4A, KMI-1303 did not show radiosensitization at a lower dose (enhancement ratio at 10% survival was 1.0) but showed a weak radiosensitization effect at the high dose, a similar tendency to the effect of *BACE1* siRNA with U2OS cells. Other BACE1 inhibitors should be tested in future studies with different types of cells.

### 3.3. Radiosensitization Effect of BACE1 Knockdown in SAOS Cells

Osteosarcoma U2OS cells are known to have wild type p53 function, whereas osteosarcoma SAOS cells are reported to possess mutant p53 status. Because, as shown in Figure 1, p53 inactivated T-Rex HeLa cells showed a higher radiosensitization effect, we examined the radiosensitization level of *BACE1* siRNA in SAOS cells. As shown in Figure 4B, *BACE1* knocked down showed a greater radiosensitization effect at higher doses than 4 Gy compared to U2OS cells with an enhancement ratio of 1.4 at 1% survival.

## 4. Discussion

In this study, we focused on the *BACE1* knockdown effect on radiosensitization in human cancer cell lines, cervical carcinoma HeLa, breast cancer MDA-MB-231, osteosarcoma U2OS and SAOS cells, and normal human lung fibroblast WI-38 cells. A slight cytotoxic effect was observed in these cells by *BACE1* knockdown alone. This cytotoxic effect was shown to be associated with apoptosis induction in U2OS cells. *BACE1* siRNA showed radiosensitization effects on HeLa and SAOS cells, which harbor p53 mutations, whereas a weaker radiosensitization effect was observed in U2OS cells, which are known to have wild type p53 function. The results possibly suggest that *BACE1* knockdown may cause radiosensitization effects more efficiently in p53 mutated cancer cells. Therefore, BACE1 functional inhibition may lead to selective radiosensitization in cancer cells. This point should be further evaluated with future studies.

BACE1 is an aspartic protease member of the peptidase A1 protein family, and it is localized mainly in the later membrane of the Golgi/trans-Golgi network but also minorly in the endoplasmic reticulum, endosome, and cell surface. *BACE1* is expressed at high levels in the brain and pancreas and is present in other tissues ubiquitously and blood plasma (for details, see GeneCards^®^, http://www.genecards.org, accessed on 29 December 2023). It is known that some fractions of cancer cells show gene amplification, mutations (Figure S1), and/or the overexpression of BACE1 (for details see Expression Atlas, http://www.ebi.ac.uk/gxa/, accessed on 30 October 2023), but the function of BACE1 or its substrate is not fully clarified in cancer cells. The radiosensitization effect may be different among the cancer cells with high and low levels of BACE1. The DEPMAP project CRISPR (DepMap Public 23Q2 + Score, Chronos, accessed on 30 October 2023) indicated that synovial sarcoma and neuroblastoma cell lines are dependent on *BACE1* expression, whereas head and neck cancers are not. RNAi (Achilles + DRIVE + Marcotte, DEMETER2) showed that hematopoietic and lymphoid cancer cells are dependent on BACE1 expression.

Several splicing variant forms are also reported for BACE1 [23]. Thus, there may be a possibility that particular isoforms of BACE1 could be involved in radiation damage response. Evaluation of the effect of the knockdown of specific isoforms may lead to selective radiosensitization by targeting radiation response-related isoforms of BACE1.

BACE1 is also recently suggested to be involved in double-strand break repair, and the knockdown of the *BACE1* gene in HeLa cells resulted in an increase in γH2AX foci in a genome-wide screening assay [21]. Because the γH2AX level is increased and G2/M arrest is slightly increased, it is speculated that BACE1 functional inhibition may radiosensitize by causing a delay in DNA damage repair. Considering its function as a peptidase, BACE1 may indirectly function in the maintenance of genomic stability.

The mechanism of radiosensitization by the functional inhibition of BACE1 should be further elucidated. It should be also studied whether BACE1 inhibition leads to a radiosensitization effect on cancer cells, which contain p53 pathway aberration caused by the mutation or dysfunction of other proteins in the pathway. It is known that more than 50% of various types of cancer cells harbor p53 mutations [24]; therefore, BACE1 inhibition may be useful for radiosensitization in various types of cancer. If the radiosensitization effect is observed widely in p53 pathway-mutated cells, this property could give an advantage to achieve a selective radiosensitization effect on cancer cells over normal cells.

Potent and specific inhibitors for BACE1 are known for the clinical treatment of Alzheimer’s disease [25].

We found that the BACE1 inhibitor KMI-1303 showed a slight radiosensitization effect in U2OS cells at a higher dose of 8 Gy. Because different types of BACE1 inhibitors have been reported, it should be necessary to examine the variety of BACE1 inhibitors with diverse time courses to evaluate their radiosensitization effects.

Because BACE1 inhibition shows little cytotoxic effect on normal cells and in the reported mouse models, this radiosensitization target may be unique. Additionally, we found that the radiosensitization effect in p53-mutated cells shows a higher radiosensitization effect at higher doses than 5 Gy. BACE1 may be useful for radiation therapy with a higher dose per fraction, such as particle beam therapy. Therefore, the effects of various types of BACE1 inhibitors on radiosensitization in cell-based and in vivo models are expected to be evaluated to achieve a better radiosensitization effect targeting BACE1.

## Data Availability

The data that supported the findings of the results are available from the corresponding author upon reasonable request.

## Supplementary Materials

The following supporting information can be downloaded at https://www.mdpi.com/article/10.3390/cimb46010028/s1, Figure S1: Genetic alteration of the *BACE1* gene in cancer cases.
