# Supplementary figures and images for: Effect of Functional Inhibition of BACE1 on Sensitization to γ-Irradiation in Cancer Cells
Source: Curr Issues Mol Biol. 2024 Jan 2;46(1):450–60. doi: 10.3390/cimb46010028 (PMC10814450; doi:10.3390/cimb46010028)

Figure S1

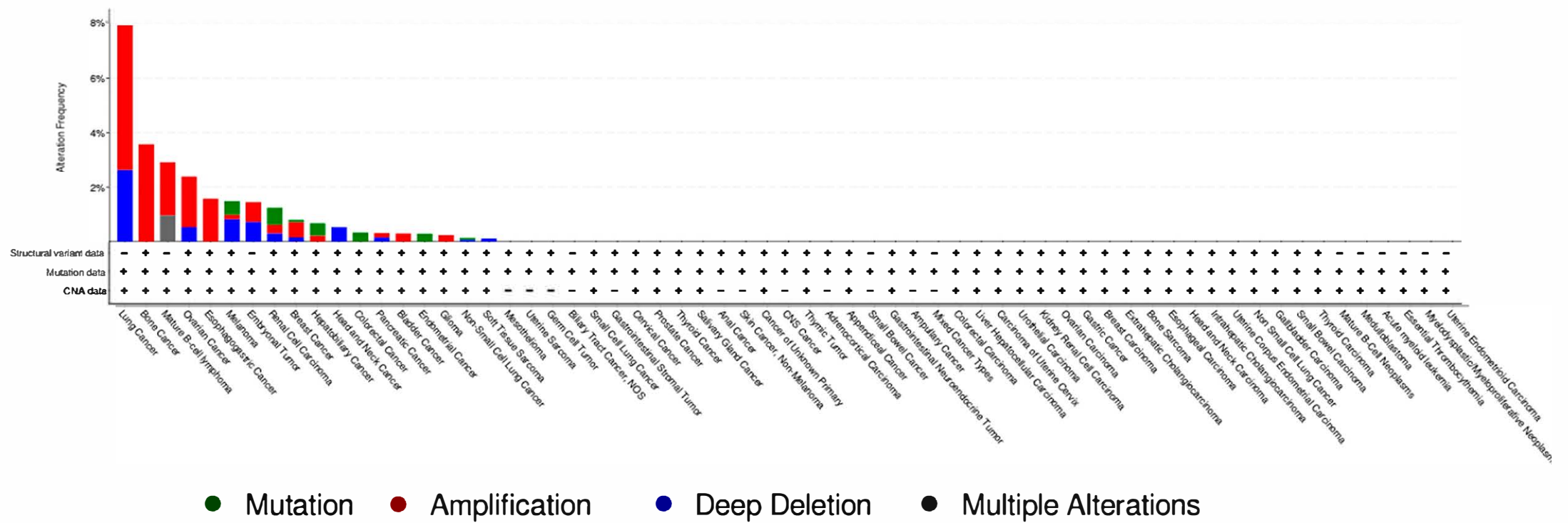

Supplement: Supplementary file 1 [file cimb-46-00028-s001.zip › cimb-2721754-supplementary.pdf]
